# Host autophagy limits *Toxoplasma gondii* proliferation in the absence of IFN-γ by affecting the hijack of Rab11A-positive vesicles

**DOI:** 10.3389/fmicb.2022.1052779

**Published:** 2022-12-01

**Authors:** Lingtao Pan, Yimin Yang, Xueqiu Chen, Mingxiu Zhao, Chaoqun Yao, Kaiyin Sheng, Yi Yang, Guangxu Ma, Aifang Du

**Affiliations:** ^1^Institute of Preventive Veterinary Medicine and Zhejiang Provincial Key Laboratory of Preventive Veterinary Medicine, College of Animal Sciences, Zhejiang University, Hangzhou, China; ^2^Department of Biomedical Sciences and One Health Center for Zoonoses and Tropical Veterinary Medicine, Ross University School of Veterinary Medicine, Basseterre, Saint Kitts and Nevis, West Indies

**Keywords:** autophagy, *Toxoplasma gondii*, proliferation, Rab11A, TgGRA2

## Abstract

**Introduction:**

Autophagy has been recognized as a bona fide immunological process. Evidence has shown that this process in IFN-γ stimulated cells controls *Toxoplasma gondii* proliferation or eliminates its infection. However, little is known about the effect of *T. gondii* infection on the host cell autophagy in the absence of IFN-γ.

**Methods:**

Multiple autophagy detection methods and CRISPR/CAS9 technology were used to study *T. gondii*-induced autophagy in HeLa and several other mammalian cell lines.

**Results:**

Here, we report increased LC3 II, autophagosome-like membrane structures, enhanced autophagic flux, and decreased lysosomes in a range of mammalian cell lines without IFN-γ treatment after *T. gondii* infection. Specifically, disruption of host *atg5* (a necessary gene for autophagy) in HeLa cells promoted the intracellular replication of *T. gondii*, with the transcript level of *rab11a* increased, compared with that in wild-type cells. Further, after *T. gondii* infection, the abundance of Rab11A remained stable in wild-type HeLa cells but decreased in *atg5*^−/−^ mutant. Disruption of *rab11a* in the HeLa cells compromised the proliferation of *T. gondii*, and increased the transcription of *gra2* in the parasite. Compared to the *T. gondii* wild-type RH∆*ku80* strain, the ∆*gra2* mutant induces enhanced host autophagy in HeLa cells, and results in slower replication of the parasite.

**Discussion:**

Collectively, these results indicate that host cell autophagy can limit *T. gondii* proliferation in an IFN-γ independent manner, possibly by affecting the hijack of host Rab11A-positive vesicles by the parasite which involved TgGRA2. The findings provide novel insights into *T. gondii* infection in host cells and toxoplasmosis research.

## Introduction

Autophagy has now been recognized as a *bona fide* immunological process in addition to its roles in controlling metabolic and intracellular biomass and organelle (Deretic, 2011). The process itself and the related proteins play a role in resistance to various pathogen infections in metazoan organisms (Levine et al., 2011). A complex and tightly regulated classical autophagy starts with the formation of a double-membrane phagophore, which then stretches and closes to form an autophagosome containing the autophagy cargoes, and is ultimately converted to an autolysosome, the degradation organelle (Długońska, 2017). A key event in the maturation of autophagosomes is the conversion of LC3 from a diffuse cytosolic form (LC3 I) to a lipidated form (LC3 II) which requires the autophagy-related protein ATG5 (Jiang and Mizushima, 2015), and it has been shown by knocking out host cell ATG5 that autophagy limits proliferation of a variety of bacteria (Subauste, 2009). *Toxoplasma gondii*, a typical intracellular parasitic pathogen that is estimated to affect 1/3 of the world population (Hunter and Sibley, 2012; Molan et al., 2019; Dubey, 2020), can only replicate efficiently by manipulating and modifying its host cells to create a parasitophorous vacuole (PV) (Blader and Koshy, 2014; Hakimi et al., 2017; Mordue and Hunter, 2020), which can be a perfect target for host autophagy. It has been reported that autophagy plays a potential role in limiting the proliferation of a variety of intracellular pathogens including *Toxoplasma gondii* (Halonen, 2009; Subauste, 2009; Ghartey-Kwansah et al., 2020; Wu et al., 2020). There has been a view that modulating autophagy may become a new strategy against toxoplasmosis (Cheng et al., 2022).

In human infections, the CD40-CD40L interaction between activated T cells and macrophages helps eliminate *T. gondii* parasitizing phagocytes through autophagy (Deretic et al., 2013), and a non-classical autophagy pathway that relies on ubiquitination was found to restrict *T. gondii* growth by targeting the PV in IFN-γ-treated HeLa cells (Selleck et al., 2015). In the case of murine infection with *T. gondii*, the IFN-γ inducible immunity-related GTPases (IRGs) and guanylate-binding proteins (GBPs) are key factors in the host’s clearance of avirulent Type III *T. gondii* (Zhao et al., 2009; Khaminets et al., 2010), and a core set of autophagy proteins is required for this mechanism although the targeted PV does not appear to be fused with endosomes or lysosomes (Selleck et al., 2015). Roughly speaking, in studies exploring the role of host cell autophagy in resistance to *T. gondii* infection, most researchers found or considered IFN-γ as the key inducer of immune response in the early stage of infection, such that *T. gondii*-induced autophagy occurs only in the presence of IFN-γ stimulation and targets the parasite PV whether or not the lysosomes are involved (Deretic, 2011; Selleck et al., 2013; Ohshima et al., 2014; Park et al., 2016; Latré et al., 2017).

Other researchers, however, found that *T. gondii* infection can also induce host cell autophagy in the absence of IFN-γ, but it does not limit the parasite proliferation, and the autophagy products might instead be exploited by the parasite (Wang et al., 2009; Gao et al., 2014). Considering that *T. gondii* is auxotrophic for many metabolites which results in utilizing host cell organelles and sequestering Golgi-derived Rab- GTPases-positive vesicles into the PV (Coppens et al., 2006; Romano et al., 2013, 2017; Coppens, 2017), the parasite likely needs the autophagy products for its rapid proliferation. However, a consensus has not yet been reached on whether *T. gondii* induces host autophagy in an IFN-γ dependent or independent manner, particularly little is known about the effect and mechanism of autophagy on/in the parasite infection in host cells without IFN-γ stimulation.

We hypothesized that autophagy in host cells can be induced by *T. gondii* infection in an IFN-γ independent manner, which can also play a role in limiting the proliferation of the parasite. To verify this hypothesis, autophagy in a range of mammalian cells that were not treated with IFN-γ and challenged with different strains of *T. gondii* was determined in a variety of ways, and its effect on the proliferation of the parasite and the mechanisms have also been investigated. Now exciting findings on autophagy induced by *T. gondii* infection in unstimulated host cells and a better understanding of the mechanism affecting the parasite have been obtained.

## Materials and methods

### Cell lines and parasite strains

The HeLa, HFF-1, HEK293T, and Vero cells were obtained from the Cell Bank of the Chinese Academy of Sciences (Beijing, China), cultured in complete high glucose DMEM (Biological Industries, Israel) supplemented with 10% (15% for HFF-1) fetal bovine serum (FBS; Gibco, South America) and 100 U/mL penicillin plus 100 μg/mL streptomycin (Gibco, USA) at 37°C with 5% CO_2_ (Zhuang et al., 2020). HeLa cell *atg5*^−/−^ and *rab11a*^−/−^ mutants were cultured under the same conditions as the wild-type cells. The *T. gondii* type I strain RHΔ*ku80* and type II strain ME49 were preserved in our lab. The tachyzoites used in all experiments were propagated in HFF-1 cells cultured in the same medium but with 2.5% FBS. Before infection, parasites were scraped, purified, and counted as described previously (Shen et al., 2017).

### Reagents and antibodies

Hoechst 33342 and Lyso-Tracker Red were purchased from Beyotime Biotechnology (Shanghai, China); Lipofectamine 2000 from Thermo Fisher Scientific (Shanghai, China); Bafilomycin A1 and Torin1 from Selleck Chemicals (Houston, USA); Puromycin from MedChemExpress (New Jersey, USA); Adenovirus Ad-mRFP-GFP-LC3 and Ad-GFP-LC3 from HANBIO (Shanghai, China); polybrene from Sigma-Aldrich (Saint Louis, USA); Anti-Actin, Anti-LC3, Anti-ATG5, Anti-Rab11A, and Anti-Rab18 monoclonal antibodies from Abcam (Shanghai, China); Anti-β-Tubulin polyclonal antibodies, goat anti-mouse and goat anti-rabbit HRP-conjugated Ig G (H + L) from FdBio Science (Hangzhou, China); ChamQ SYBR qPCR Master Mix (High ROX Premixed) from Vazyme (Nanjing, China); Protease inhibitor cocktail (Bimake, Texas, USA); TRIzol reagent and Goat anti-Rabbit IgG (H + L) Highly Cross-Adsorbed Secondary Antibody, Alexa Fluor™ Plus 594, from Invitrogen (Shanghai, China); ReverTra Ace qPCR RT Kit from TOYOBO (Oosaka, Japan).

### Western blot

Cells were harvested and suspended in ice-cold radioimmunoprecipitation assay buffer (RIPA) lysis buffer supplemented with a protease inhibitor cocktail, and incubated for 30 min on a horizontal shaker at 50 rpm under 4°C (Zhuang et al., 2020). Some protein samples were extracted with TRIzol reagent, resuspended in 1% SDS solution, and boiled in SDS loading buffer afterwards as described previously (Simões et al., 2013; Wen et al., 2020). The soluble protein samples after 12,000 g × 10 min centrifugation were boiled for 10 min in SDS loading buffer, separated by SDS-PAGE, and transferred to 0.22 μm PVDF membrane (Millipore, USA). Membranes were blocked in 5% no-fat milk in Tris-buffered saline (TBS) supplemented with 0.5% Tween 20 for 2 h at 37°C. The incubation of primary antibodies in primary antibody dilution buffer took overnight at 4°C. The primary antibodies (1:5000) were detected using goat anti-mouse HRP-conjugated Ig G (H + L) (1:5000) or goat anti-rabbit HRP-conjugated Ig G (H + L) (1,5,000). Signals were documented by the ChemiDoc™ chemiluminescence system (BioRad, USA) after membranes were subjected to ECL substrates (FdBio Science, China) after fully rinsing. No image was changed in brightness and/or contrast. Densitometric analysis was measured by Image J (1.52v, National Institutes of Health, USA).

### Electron microscopy

Cells cultured in T25 cell flasks (Thermo, USA) were randomly divided into two groups, the negative control group, and *T. gondii* infection group. The cells in the latter were infected by purified tachyzoites at an MOI of 2:1. 24 h post-infection, cells in both groups were fixed with 2.5% glutaraldehyde for at least 24 h followed by three washed (15 min each) in phosphate-buffered saline (PBS) to remove residual glutaraldehyde. Afterwards, they were embedded in agar and then stained in osmium acid for 1 h. The samples were dehydrated in gradient ethanol (30, 50, 70, 80%) and gradient acetone (90, 95%), each solution for 15 min after being washed three times in PBS. At the end of dehydration, the samples were treated with absolute acetone twice, 20 min each. The dehydrated samples were placed sequentially in a mixture of acetone and the final Spurr resin (1:1), acetone and the final Spurr resin (1:3), and the pure final Spurr resin for 1, 3, and 12 h at room temperature, respectively. Specimens were then placed in 0.2 mL centrifuge tubes containing Spurr resin and heated at 70°C for more than 9 h. Afterwards, they were sectioned in LEICA EM UC7 ultratome (Leica Microsystems, Germany). The ultra-thin sections were stained with uranyl acetate and alkaline lead citrate for 5 to 10 min, respectively, and were observed by a Hitachi Model H-7650 TEM (Hitachi, Japan).

### Transfection and cell staining

Two-color fluorescent adenovirus *Ad-mRFP-GFP-LC3* and single-color fluorescent adenovirus *Ad-GFP-LC3* (Wang et al., 2017; Yu et al., 2017) were diluted to 5 × 10^4^ TU/mL in DMEM and used to infect HeLa, HFF-1 and Vero cells grown in the laser confocal Petri dishes (Corning, USA) for 2 h. 24 h later, the cells were randomly divided into negative control group and the infection group. The infection group cells were infected with *T. gondii* tachyzoites at an MOI of 2:1.

All cells infected with *Ad-mRFP-GFP-LC3* were fixed with 4% paraformaldehyde at 24 h post-infection and visualized under an LSM880 confocal microscope (Carl Zesis AG, Germany). The Torin1 treated Vero cells were incubated in culture medium supplemented with 0.25 μM Torin1 (Granatiero et al., 2021) whereas the two other group Vero cells were incubated in culture medium supplemented with the same volume of DMSO (the solvent for Torin1) 6 h before fixed. All cells infected with *Ad-GFP-LC3* were incubated in normal culture medium supplemented with 75 nM Lyso-Tracker Red and 0.5 μg/mL Hoechst 33342 for 25 min at 23 h 30 min post-infection. These cells in the laser confocal Petri dishes were directly visualized under the confocal microscope after being rinsed in fresh culture medium.

The quantification of fluorescence was performed following this paper (Jesen, 2013).

### Gene disruption

HeLa *atg5*^−/−^ cells and *rab11a*^−/−^ cells were generated by disrupting the corresponding gene loci using Lenti-CRISPR/CAS9 (Shalem et al., 2014). The sgRNAs were designed on http://www.e-crisp.org/E-CRISP/designcrispr.html, ligated to *BsmB* I (NEB, USA) digested *LentiCRISPR v2-gDNA* plasmids with T4 ligase (TAKARA, Japan). Lentivirus was produced by transfecting 8 × 10^5^ HEK293T cells with 1 μg *LentiCRISPR v2-GOI* plasmid, 0.75 μg packaging plasmid *psPAX2* and 0.25 μg envelop plasmid *pMD2.G* premixed in 200 μL DMEM supplemented with 10 μL Lipofectamine 2000. Cell cultures containing lentiviruses were collected 48 h after transfection and supplemented with 6 μg/mL polybrene. HeLa cells in 24-well plates were incubated with the lentiviruses for 12 h and were further cultured for an additional 36 h in fresh culture medium before 1 μg/mL puromycin screening. After two rounds of screening, cells were seeded into 96-well plates by limiting dilution and cultured in DMEM supplemented with 20% FBS. Deletion of ATG5 and Rab11A were confirmed by Western Blot Analysis.

The gene editing procedure for *T. gondii* was described previously (Shen et al., 2017).

All sgRNAs and primers are listed in Supplementary Table 1.

### Intracellular replication assay

To examine the intracellular replication of *T. gondii*, HeLa cells were seeded on cell slides in 24-well plates, infected with tachyzoites for 2 h, washed three times with PBS, and then cultured with fresh medium for an additional 22 h. Cells were fixed with methanol or 4% paraformaldehyde, and intracellular parasites were labeled with rabbit anti-TgGAP45 antibodies. Goat anti-rabbit IgG antibodies conjugated to Alexa Fluor 594 were used as indicated. The number of parasites per PV was determined by manual counting using a fluorescence microscope as 2 when the visible number was 2, 4 when the visible number was 3 to 4, 8 when the visible number was 5 to 8, or ≥16 when the visible number was over 9. Data were collected from at least 150 vacuoles on three individual coverslips (Selleck et al., 2015).

### RNA extraction and real-time quantitative PCR

Total RNAs were extracted using TRIzol reagent, and immediately reverse-transcribed into cDNA by ReverTra Ace qPCR RT Kit. The cDNA samples were diluted to an appropriate concentration. The real-time quantitative PCR was performed with ChamQ SYBR qPCR Master Mix (High ROX Premixed) on the LightCycle 480 system (Roche).

### Statistical analysis

All quantifiable data were analyzed by GraphPad Prism 8.0. When it came to difference analysis, the 2-tailed Student’s t-test was carried out with means ± standard error [SEM]. *p*-values smaller than 0.05 were considered statistically significant.

## Results

### *Toxoplasma gondii* infection increases LC3 II and autophagosomes in unstimulated cells

**Figure 1:**
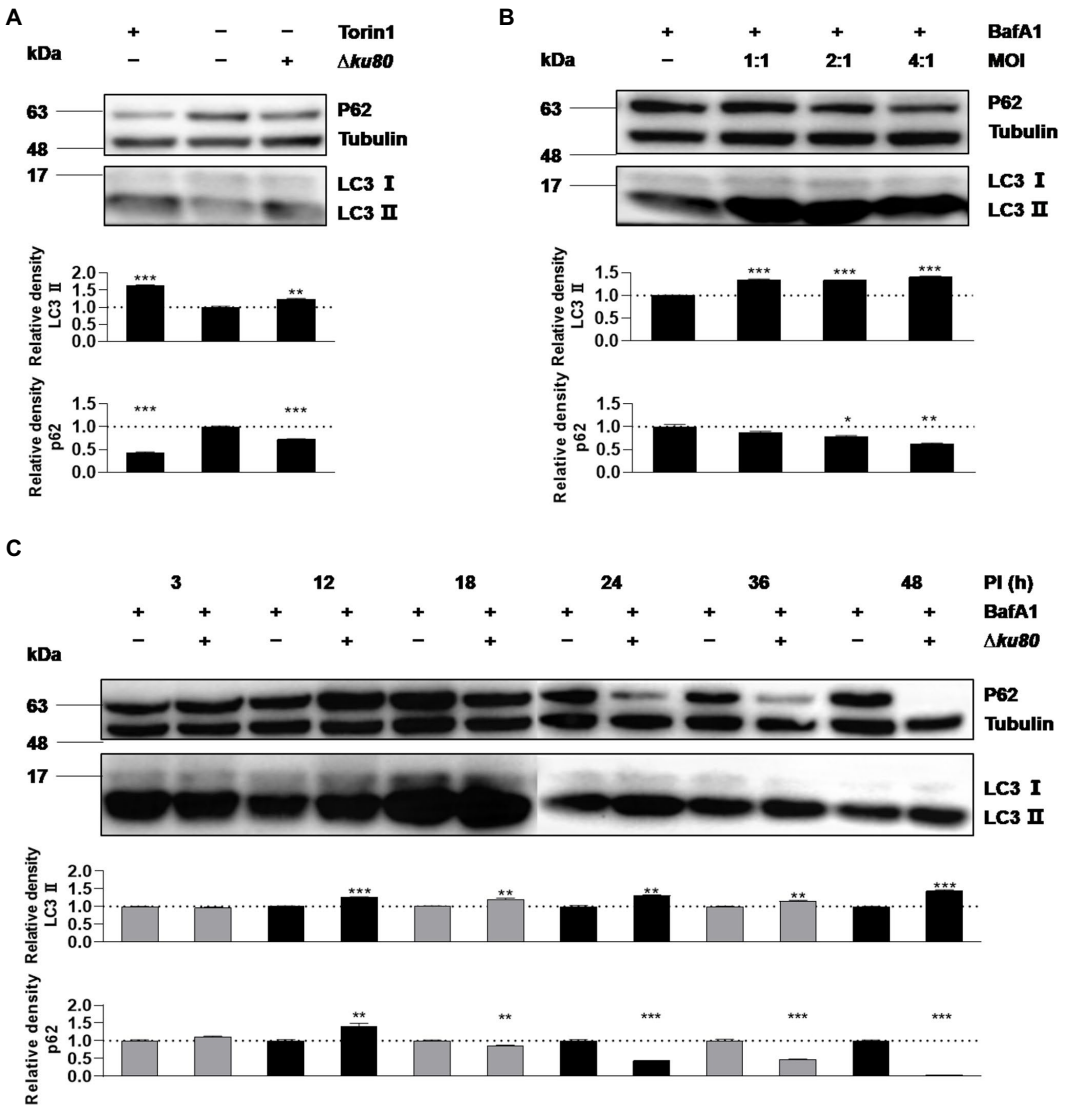
*Toxoplasma gondii* infection increases LC3 II in host cells. **(A)** HeLa cells from blank control group, autophagy inducer Torin1 (0.25 μM) treated group, and *T. gondii* RH∆*ku80* strain challenged group (∆*ku80*) were collected, and proteins of equal amounts of cell lysates were separated on 15% SDS-PAGE gels. LC3 (16 ~ 18 kDa) and p62 (~62 kDa) were detected using rabbit anti-LC3 monoclonal antibody and rabbit anti-p62 monoclonal antibody, and Tubulin (~55 kDa) was detected using mouse anti-β-tubulin polyclonal antibodies as a loading control. **(B)** HeLa cells challenged by *T. gondii* tachyzoites at different multiplicities of infection (MOI) were treated with lysosomal inhibitor BafA1 (10 nM) for 2 h starting at 22 h post-infection, Western blot analysis was the same as **(A, C)** Hela cells challenged by *T. gondii* at an MOI of 2:1 for different durations of infection (DOI) were harvested and prepared, Western blot analysis was the same as **(A)**. Relative densities of LC3 II/p62 were the ratios of the density of LC3 II/p62 to the density of Tubulin of the same individual preparations. The relative densities of the negative controls were set as one arbitrary unit whereas those of the treated groups were ratios to the negative controls. Each value was the mean ± SEM of three measurements (*p ≤ 0.05; **p ≤ 0.01; ***p ≤ 0.001; *t*-tests).

Without stimulation by IFN-γ, autophagy in HeLa cells induced by *T. gondii* infection was firstly determined by detecting LC3 (Figures 1A,B), as the conversion from LC3 I to LC3 II indicates the formation of autophagosomes. In HeLa cells challenged with *T. gondii* RH∆*ku80* tachyzoites, we found that the relative density of LC3 II was 1.25 times (*p* < 0.001) of that of the negative control, whereas it was 1.65 times (*p* < 0.001) in the positive control cells treated with 0.25 μM Torin1, an ATP-competitive MTOR inhibitor that induces autophagy in mammalian cells (Thoreen et al., 2009)(Figure 1A). In contrast to increased levels of LC3 II, p62 decreased in the *T. gondii* infected HeLa cells (Figure 1A), further verifying that *T. gondii* infection induces autophagic activities in host cells even without IFN-γ stimulation, as depletion of p62 occurs in autophagy (Jiang and Mizushima, 2015). Since lysosomal turnover but not a simple cellular level of endogenous LC3 is the marker for autophagy (Tanida et al., 2005), to verify whether increased levels of LC3 II may be due to increased autophagy, or decrease in degradation, Bafilomycin A1 (BafA1), a lysosomal inhibitor was introduced to treat cells for 2 h before their harvest. In BafA1 treated cells, LC3 II was increased by 35% at the MOI of 1:1 (*p* < 0.001), and with no further increase detected at the MOI of 2:1 and 4:1 (Figure 1B), suggesting that autophagic activities were induced by *T. gondii* infection in cells that were not stimulated by IFN-γ. And although the increase of LC3 II was not detected in unstimulated HeLa cells at 3 h post-infection with *T. gondii*, a 25–65% increase (*p* < 0.0001) was detected from 12 h to 48 h post-infection (Figure 1C). In addition to HeLa cells, consistent results were obtained in HFF-1, HEK293T, and Vero cells infected with *T. gondii* RH∆*ku80* strain, as well as with type II ME49 strain (see Supplementary Figure S1).

**Figure 2:**
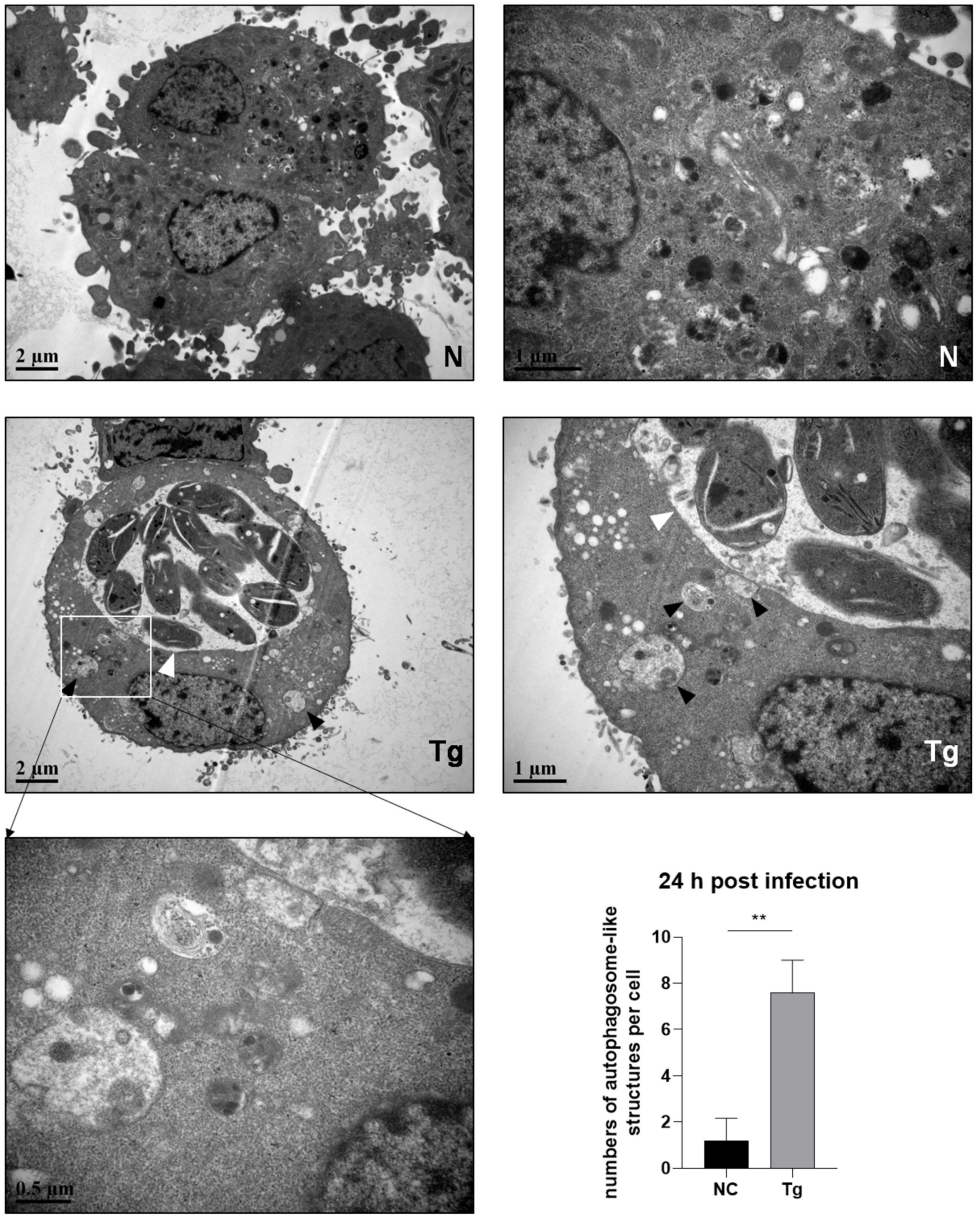
Autophagosome-like membrane structure increases in *T. gondii* infected cells. HeLa cells were challenged by *T. gondii* RH∆*ku80* tachyzoites at an MOI of 2:1 and were fixed with 2.5% glutaraldehyde at 24 h post-infection. Autophagosome-like membrane structures (As the black arrows show) could be observed in most of the cells with *T. gondii* PV (As the white arrows show) (Tg), which did not exist in negative control group cells (N). The numbers of autophagosome-like structures in both groups were counted. Each value is the mean ± SEM of 5 cells (**p ≤ 0.01; *t*-tests).

Since the maturation of autophagosomes was indicated by the conversion from LC3 I to LC 3 II and decreased p62, we observed untreated and *T. gondii* infected HeLa cells by electron microscopy. Autophagosome-like membrane structures (The black arrows shown) were observed in infected HeLa cells (with parasite PVs shown by the white arrows), while no or few of these structures were observed in untreated cells (Figure 2). Autophagosome-like membrane structures were also observed in *T. gondii* infected Vero and HFF-1 cells (Supplementary Figure S2). These results clearly showed that *T. gondii* infection induces autophagy in unstimulated mammalian cells.

### *Toxoplasma gondii* infection promotes autophagic flux in unstimulated cells

**Figure 3:**
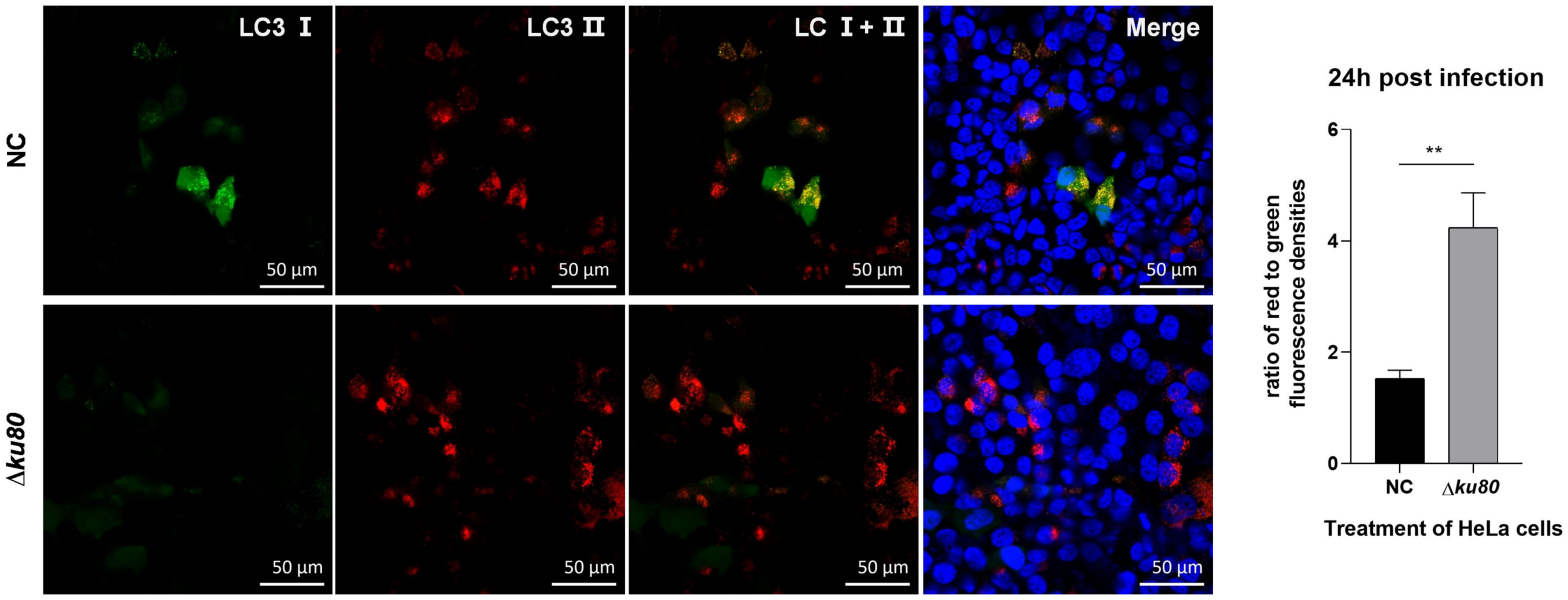
Activated autophagic flux in *T. gondii* infected cells. HeLa cells overexpressing mRFP-GFP-LC3 were challenged by *T. gondii* RH∆*ku80* tachyzoites at an MOI of 2:1, and fixed with 4% paraformaldehyde at 24 h post-infection. Nuclei were stained with 4=,6-diamidino-2-phenylindole (DAPI). The fluorescence signals in *T. gondii* infected cells (∆*ku80*) and uninfected cells (N) were collected in exactly the same way. Quantitative analysis of fluorescence was done using Image J. The mean density of single fluorescence over the full image range of each merged image was measured after the channels splitting, each value is the mean ± SEM of 4 microscope fields (**p ≤ 0.01; *t*-tests).

Based on the *T. gondii* infection-induced LC3 II accumulation and autophagosome maturation, autophagic flux in HeLa cells was analyzed. Overexpression of the dual-color fluorescent protein mRFP-GFP-LC3 was achieved by adenovirus infection. In these adenovirus-infected cells, the increased ratio of red to green fluorescence indicates the increased autophagic flux. In HeLa cells overexpressing mRFP-GFP-LC3, compared to the uninfected cells, a slightly stronger red fluorescence and almost no green fluorescence were observed in RH∆*ku80* infected cells at 24 h post infection (Figure 3). The statistical results showed that the ratio of red to green fluorescence in the *T. gondii* infected group cells was significantly higher (*p* < 0.01) than that of the uninfected group cells. These results suggested that *T. gondii* infection promoted autophagic flux in unstimulated HeLa cells. Increased autophagic flux was also observed in HFF-1 cells (Supplementary Figure S3).

In conclusion, *T. gondii* infection induces enhanced autophagic activities in host cells without IFN-γ stimulation and leads to the formation of autophagolysosomes.

### LC3 II and lysosomes are partially recruited to the *Toxoplasma gondii* PV

**Figure 4:**
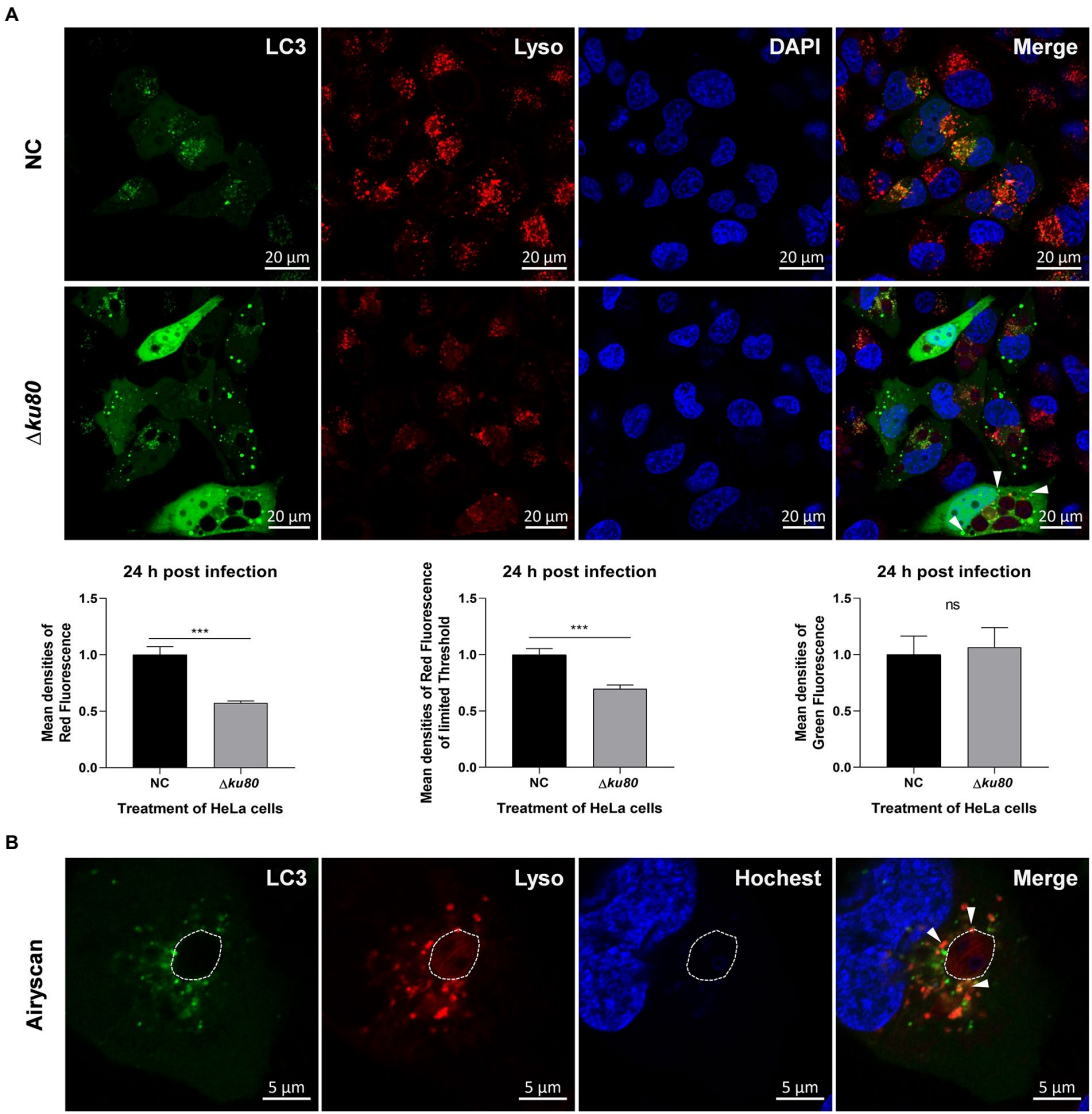
The distribution of lysosomes in *T. gondii* infected HeLa cells. **(A)** HeLa cells overexpressing EGFP-LC3 were challenged by *T. gondii* RH∆*ku80* tachyzoites at an MOI of 2:1, and live cells were stained at 24 h post-infection. The nuclei were stained with Hoechst and lysosomes with Lyso-Tracker Red. The fluorescence signals were collected within 40 min after staining (∆*ku80*). N: uninfected control cells. Quantitative analysis of fluorescence was done using Image J. Mean density of single fluorescence of each merged image was measured after channels splitting, each value is the mean ± SEM of 6 microscope fields (***p ≤ 0.001; t-tests). **(B)** The parasite PVM was outlined with a dashed line, and LC3 recruited to the PVM was indicated by arrows.

Fusion of the pathogen-containing endosomes with lysosomes is a critical effector mechanism that acts as the starting point for killing pathogens (Długońska, 2017). As shown above, autophagolysosomes are formed in host cell autophagy induced by *T. gondii* infection, leading to the speculation that the fusion of autophagosomes with lysosomes, in this case, may act as a similar role in an anti-parasite mechanism. Therefore, the distribution of LC3 and lysosomes were observed in Hela cells. Overexpression of single-color fluorescent protein EGFP-LC3 was achieved by adenovirus infection and lysosomes were stained in living cells by Lyso-Tracker Red. Compared to uninfected cells, the green fluorescence of LC3 in RH∆*ku80* infected HeLa cells was slightly enhanced (*p* > 0.05) with more punctate distributions, while the red fluorescence of lysosomes was significantly decreased (*p* < 0.001) (Figure 4A).

The decreased densities of red fluorescence in HeLa cells indicated the consumption of lysosomes in content digestion of autophagy, but only a small amount of LC3 and lysosomes were localized on the parasite PVM (shown by white arrows) with most distributed close to the PV (Figure 4B). These results indicated that *T. gondii* infection induces autophagy in HeLa cells without IFN-γ stimulation, and lysosomes are involved. The punctate aggregation of LC3 that does not localize on the parasite PVM might suggest that this autophagy not only takes place at the parasite PV, which indicates a different mechanism from pathogen killing by endolysosomes.

### Autophagy in unstimulated cells restricts *Toxoplasma gondii* proliferation

**Figure 5:**
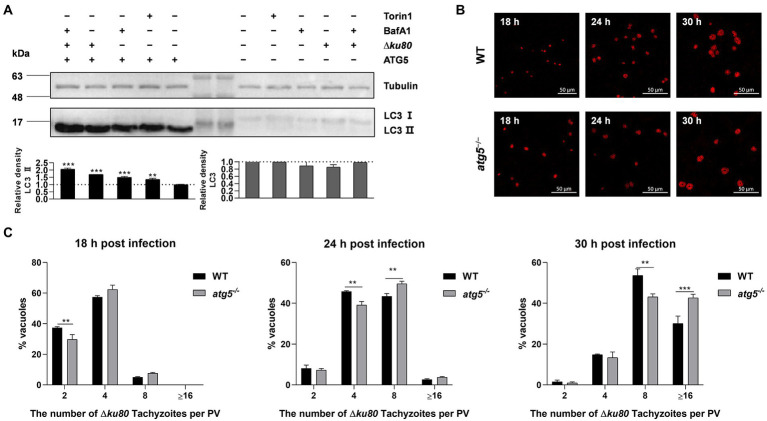
Disruption of ATG5 enhances *T. gondii* proliferation *in vitro*. **(A)** Wild-type and *atg5*^−/−^ HeLa cells were harvested and prepared at 24 h post-infection. Proteins of equal amounts of cell lysates were separated on 15% SDS-PAGE gels. LC3 (16 ~ 18 kDa) was detected using rabbit anti-LC3 monoclonal antibody, and Tubulin (~55 kDa) expression was detected using mouse anti-β-tubulin polyclonal antibodies as a loading control. Relative densities of whole LC3 or LC3 II were the ratios of the density of whole LC3 or LC3 II to the density of Tubulin. The relative densities of the negative controls were set as one arbitrary unit whereas those of the treated groups were ratios to the negative controls. Each value was the mean ± SEM of three measurements (**p ≤ 0.01; ***p ≤ 0.001; *t*-tests) **(B)** Wild-type and *atg5*^−/−^ HeLa cells were challenged by *T. gondii* RH∆*ku80* tachyzoites at an MOI of 2:1, and fixed with methanol at 18, 24 and 30 h post-infection. Rabbit anti-TgGAP45 antibodies were used to label the parasite tachyzoites. **(C)** Replication of parasites was determined by the proportion of vacuoles containing 2, 4, 8, and 16 or more tachyzoites. Each value is the mean ± SEM of three counts (**p ≤ 0.01; ***p ≤ 0.001; two-way ANOVA).

To determine the effect of *T. gondii*-induced autophagy on *T. gondii* proliferation, the autophagy-related gene *atg5* was disrupted in HeLa cells for ATG5 is necessary for autophagy (Hanada et al., 2007; Liu et al., 2015a; Supplementary Figure S4A). In *atg5*^−/−^ HeLa cells, the LC3 II formation was significantly inhibited, resulting in the levels of LC3 II below detection by Western blot with a concomitant accumulation of LC3 I (Figure 5A), which means the disruption of autophagy.

*Toxoplasma gondii* proliferation was determined by intracellular replication assays. Growth of RH∆*ku80 in vitro* normally proceeds by endodyogeny, so that at 24 h post-infection, each PV contains 1 to 16 tachyzoites. PVs containing different numbers of tachyzoites in wild-type and *atg5*^−/−^ HeLa cells at 18, 24, and 30 h post-infection were determined. Data clearly showed that proportions of PVs with 8 or ≥16 tachyzoites in *atg5*^−/−^ cells at 24 or 30 h PI were significantly higher than those of parental controls, at *p* < 0.01 and *p* < 0.001, respectively (Figures 5B,C), which indicates that the host cell autophagy induced by *T. gondii* infection restricts the parasite proliferation.

### Autophagy deficiency leads to altered Rab11A abundance in HeLa cells

More targets of *T. gondii*-induced autophagy need to be identified to reveal this additional host resistance mechanism to parasitic infection. We hypothesized that it interferes with the nutrient uptake of the parasite. Vesicles controlled by host Rabs are known to be important for *T. gondii* to obtain nutrients from host cells (Romano et al., 2013, 2017). Therefore, the Rabs-positive vesicles may be targets of autophagy induced by *T. gondii* infection.

**Figure 6:**
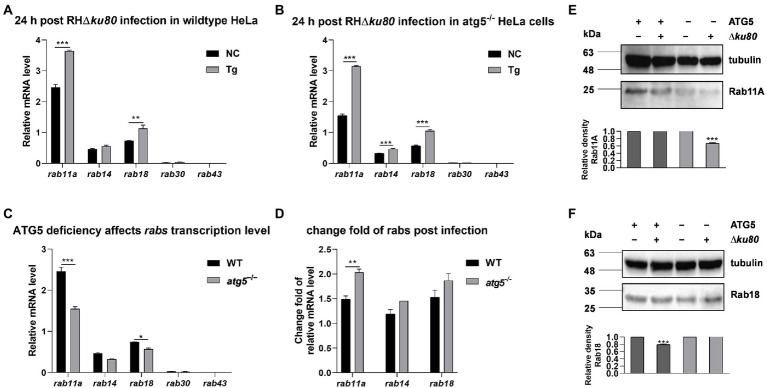
Transcripts and proteins of *rabs* in wild-type and *atg5*^−/−^ HeLa cells. HeLa cells were challenged by *T. gondii* RH∆*ku80* tachyzoites at an MOI of 2:1, and total RNAs and proteins were extracted 24 h post-infection. The relative mRNA abundance of *rabs* was quantified by qPCR, *tubulin* was used as a standard to normalize all data by 2^−ΔΔCT^ methods. Each value is the mean ± SEM of three experiments (*p ≤ 0.05; **p ≤ 0.01; ***p ≤ 0.001; *t*-tests). **(A)** The Relative mRNA abundance of *rab14*, *rab11a, rab30, rab43,* and *rab18* in untreated and *T. gondii* infected wild-type HeLa cells at 24 h post-infection. **(B)** The relative mRNA abundance of *rab14*, *rab11a, rab30, rab43,* and *rab18* in untreated and *T. gondii* infected *atg5*^−/−^ HeLa cells at 24 h post-infection. **(C)** The relative mRNA abundance of *rab14*, *rab11a*, *rab30*, *rab43,* and *rab18* in untreated wild-type and *atg5*^−/−^ HeLa cells at 24 h post-infection. **(D)** Fold changes of the transcription of *rab11a, rab14, and rab18* due to *T. gondii* infection in wild-type and *atg5*^−/−^ HeLa cells at 24 h post-infection. (E & F) Untreated and *T. gondii* infected (MOI 2:1) wild-type and *atg5*^−/−^ HeLa cells were harvested at 24 h post-infection, and proteins of equal amounts of cell lysates were separated on 15% SDS-PAGE gels. Rab11A (~24 kDa) and Rab18 (~23 kDa) were detected using rabbit anti-Rab11A and anti-Rab18 polyclonal antibodies. The relative densities of Rab11A or Rab18 were the ratios of the densities of Rab11A or Rab18 to the densities of Tubulin. The relative densities of the uninfected cells were set as one arbitrary unit whereas those of the treated groups were ratios to the negative controls. Each value was the mean ± SEM of three measurements (***p ≤ 0.001; *t*-tests).

The expression of several *rabs* at the transcriptional level in wild-type and *atg5*^−/−^ HeLa cells were examined by real-time quantitative PCR. Data showed an up-regulation of *rab11a* and *rab18* transcription at 24 h post-*T. gondii* infection in both wild-type and *atg5*^−/−^ HeLa cells, while the transcription of *rab14* was significantly increased only in *atg5*^−/−^ cells (Figures 6A,B). On the other hand, without *T. gondii* infection, the absence of ATG5 resulted in reduced transcription of *rab11a* and *rab18* in HeLa cells (Figure 6C). Compared with wild-type cells, the up-regulation of *rab11a* transcription was about 50% greater in ATG5 deficient mutant after *T. gondii* infection (Figure 6D). We further detected the abundance of Rab11A and Rab18 in uninfected and infected cells by western blot analysis. In both wild-type and *atg5*^−/−^ HeLa cells, inconsistent with the up-regulation of transcription, the protein abundance of Rab11A was not increased. It was even about 33% decreased in *atg5*^−/−^ cells after *T. gondii* infection (Figure 6E). While Rab18 did not show such a clear contrast (Figures 6D,F). In conclusion, the infection of *T. gondii* resulted in up-regulation of host cell *rab11a* transcription, and the Rab11A abundance remains unchanged in wild-type HeLa cells while decreased in autophagy deficiency mutant.

### Deletion of Rab11A aggravates *Toxoplasma gondii*-induced LC3 II accumulation and limits *Toxoplasma gondii* proliferation

**Figure 7:**
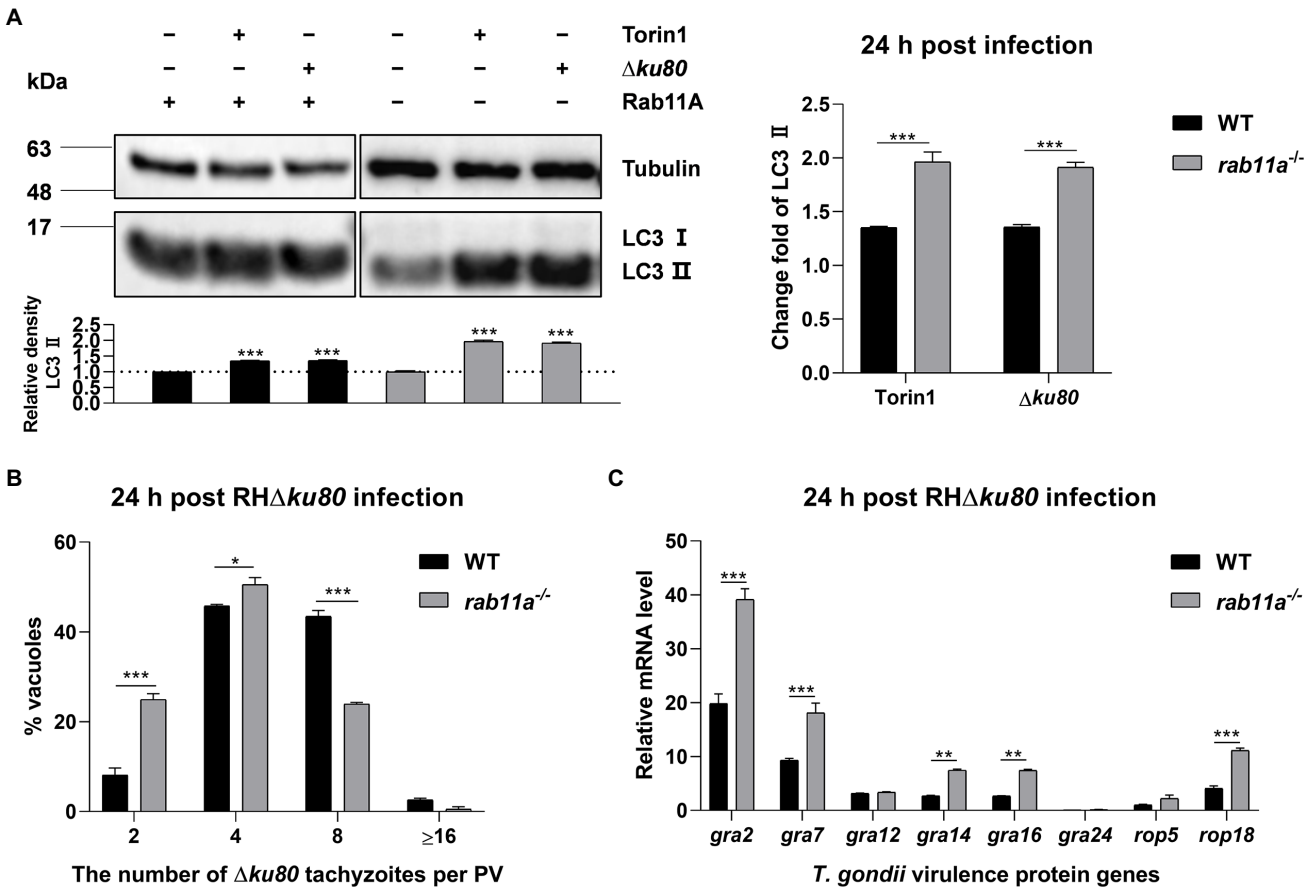
Rab11A deficiency aggravates *T. gondii*-induced host cell autophagy and limits *T. gondii* replication. **(A)** Wild-type and *rab11a*^−/−^ HeLa cells were challenged by *T. gondii* RH∆*ku80* tachyzoites at an MOI of 2:1, and cells were harvested and prepared at 24 h post-infection. Proteins of equal amounts of cell lysates were separated on 15% SDS-PAGE gels. LC3 (16 ~ 18 kDa) and p62 (~62 kDa) were detected using rabbit anti-LC3 monoclonal antibody and rabbit anti-p62 monoclonal antibody, and Tubulin (~55 kDa) was detected using mouse anti-β-tubulin polyclonal antibodies as a loading control. Relative densities of LC3 II were the ratios of the densities of LC3 II to the densities of Tubulin. The relative densities of the negative controls were set as one arbitrary unit whereas those of the treated groups were ratios to the negative controls. The fold changes of LC3 II protein abundance were the ratios of relative densities of LC3 II of infected cells to relative densities of LC3 II of untreated cells. Each value is the mean ± SEM of three measurements (***p ≤ 0.001; *t*-tests). **(B)** Replication of *T. gondii* RH∆*ku80* in wild-type and *rab11a*^−/−^ HeLa cells was determined by the proportion of vacuoles containing 2, 4, 8, and 16 or more tachyzoites. Each value is the mean ± SEM of three counts (**p ≤ 0.01; ***p ≤ 0.001; two-way ANOVA). **(C)** HeLa cells were challenged by *T. gondii* RH∆*ku80* tachyzoites at an MOI of 2:1, total RNA and protein were extracted using TRIzol at 24 h post-infection. The relative mRNA abundance of *rabs* was quantified by qPCR, *tubulin* was used as a standard to normalize all data by 2^−ΔΔCT^ methods. Each value is the mean ± SEM of three experiments (*p ≤ 0.05; **p ≤ 0.01; ***p ≤ 0.001; *t*-tests).

To determine whether Rab11A is involved in host cell autophagy induced by *T. gondii* infection, we knocked it out in HeLa cells (Supplementary Figure S5A). LC3 was detected by Western blot analysis, and the results showed that *T. gondii* infection could also cause LC3 II accumulation in *rab11a*^−/−^ HeLa cells (Figure 7A). Further analysis found that at 24 h post-infection, the relative densities of LC3 II in *rab11a*^−/−^ HeLa cells almost doubled after *T. gondii* infection, while the increase in wild-type cells was only about 35% (Figure 7A). We also investigated the effect of Rab11A deficiency on *T. gondii* proliferation. The results of intracellular replication assays showed that the parasite replication was slowed down in *rab11a*^−/−^ HeLa cells (Figure 7B). In addition, we detected the expression of various *T. gondii* secretory and transmembrane proteins at the transcriptional level in both wild-type and *rab11a*^−/−^ HeLa cells. The results showed that the deletion of Rab11A resulted in increased transcription of genes encoding dense granule proteins GRA2, GRA7, GRA14, and GRA16, and the gene coding for important virulence protein ROP18 (Figure 7C), whereas the deletion of ATG5 did not (Supplementary Figure S4B). These results indicated that Rab11A plays an important role in *T. gondii*-induced autophagy and is also important for *T. gondii* proliferation *in vitro*, as its deletion results in up-regulation of the expression of various *T. gondii* virulent proteins at the transcriptional level.

### GRA2 is involved in *Toxoplasma gondii* infection-induced host cell autophagy

GRA2 is one of the altered expressed dense granule proteins in *rab11a*^−/−^ HeLa cells. It was found to be involved in the sequestration of Rab11A vesicles by *T. gondii*, and the deletion of the protein would slightly prevent *T. gondii* from getting Rab11A (Romano et al., 2017). To investigate whether GRA2 is involved in the host cell autophagy induced by *T. gondii* infection, we knocked out *gra2* in RH∆*ku80* (Supplementary Figure S5B).

**Figure 8:**
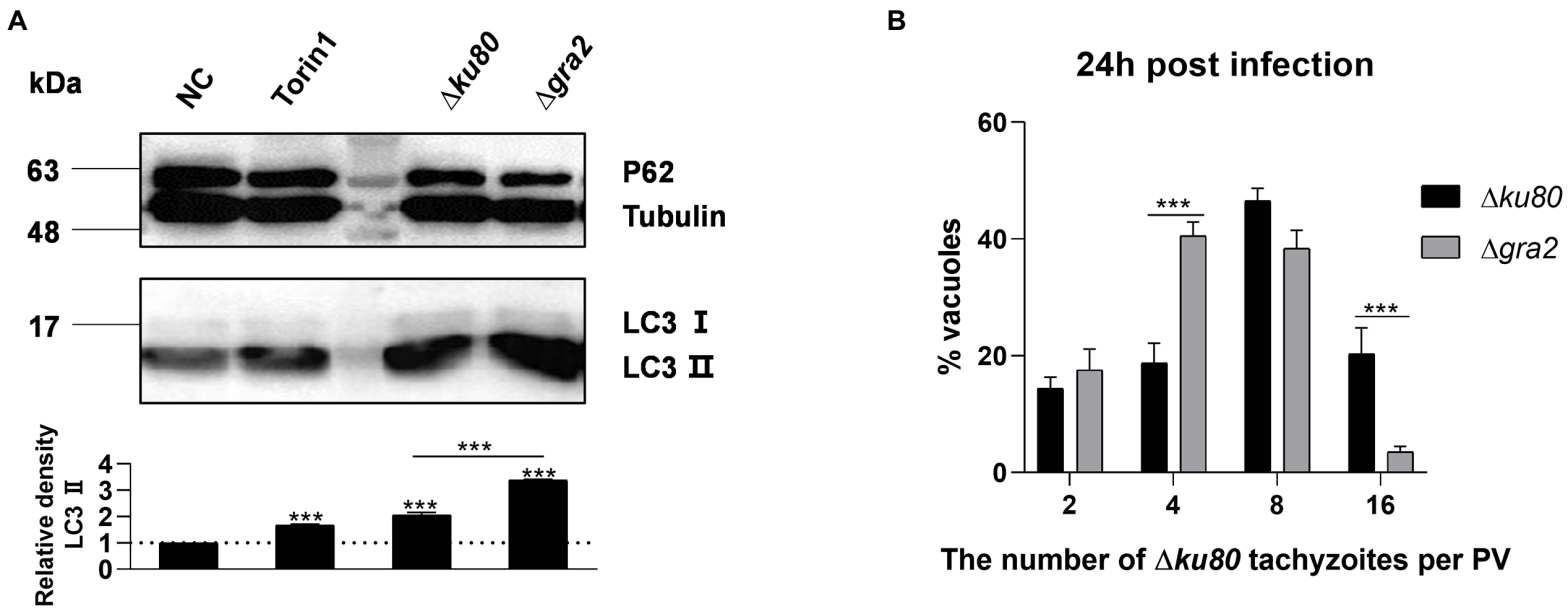
*Toxoplasma gondii* GRA2 deficiency results in greater LC3 II accumulation of the host cell and slower replication of the parasite. **(A)** HeLa cells untreated, treated by Torin1, ∆*ku80,* and ∆*gra2* infection were harvested and prepared at 24 h post-infection, and proteins of equal amounts of cell lysates were separated on 15% SDS-PAGE gels. LC3 (16 ~ 18 kDa) and p62 (~62 kDa) were detected using rabbit anti-LC3 monoclonal antibody and rabbit anti-p62 monoclonal antibody, Tubulin (~55 kDa) expression was detected using mouse anti-β-tubulin polyclonal antibodies as a loading control. Relative densities of LC3 II were the ratios of the densities of LC3 II to the densities of Tubulin. The relative densities of the negative controls were set as one arbitrary unit whereas those of the treated groups were ratios to the negative controls. Each value is the mean ± SEM of three measurements (***p ≤ 0.001; *t*-tests). **(B)** HFF-1 cells were challenged by *T. gondii* RH∆*ku80* and RH∆*ku80*∆*gra2* tachyzoites at an MOI of 2:1 and fixed by methanol at 24 h post-infection. Intracellular replication of parasites was determined by the proportion of vacuoles containing 2, 4, 8, and 16 or more tachyzoites. Each value is the mean ± SEM of three counts (***p ≤ 0.001; two-way ANOVA).

LC3 was detected in HeLa cells infected with wild-type RH∆*ku80* and ∆*gra2*, results showed that the infection with ∆*gra2* led to an approximately 140% greater increase of LC3 II in HeLa cells than that with wild-type RH∆*ku80* (Figure 8A). The effect of GRA2 deletion on the parasite proliferation was determined by intracellular replication assays. Corresponding to enhanced autophagy induced by the parasite in host cells, the intracellular replication of ∆*gra2* was slower in HFF-1 cells than in wild-type RH∆*ku80* (Figure 8B). These results indicated that GRA2 plays a role in host cell autophagy induced by *T. gondii* infection, and should be related to host Rab11A.

## Discussion

As an extremely successful parasite, *T. gondii* not only protects itself from host cytolytic factors by resisting fusion with the host degradative endolysosomal system (Clough and Frickel, 2017) but also promotes its replication by modifying its host cells (Hakimi et al., 2017). *T. gondii* infection may lead to toxoplasmosis, which poses a big threat to immunocompromised individuals with no ideal treatment. To minimize and eventually eliminate this threat, a better understanding of the interaction between parasites and host cells is needed. One keen question of ours was whether any anti-*T. gondii* mechanism in host cells is independent of IFN-γ. As far as *T. gondii* infection-induced autophagy of unstimulated host cells are concerned, possibilities are: i) *T. gondii* infection does not induce host cell autophagy; ii) *T. gondii* infection does induce host cell autophagy that either enhances or limits the parasite proliferation; iii) *T. gondii* does induce host cell autophagy but does not affect the parasite proliferation. To address these possibilities different experimental methods using a variety of experimental materials were chosen. *T. gondii* has been categorized into three clonal lineages, i.e., type I, II, and III, that differ widely in their virulence in mouse models (Liu et al., 2015b; Dardé et al., 2020). Different cell lines or different growth environments can cause differences in the degree of host cell autophagy. Our data unequivocally demonstrate that infection with *T. gondii* type I and type II strains does induce autophagy of unstimulated cells, resulting in restriction of parasite proliferation in HeLa cells. We noticed that in BafA1 treated HeLa cells, the degradation of LC3 II and 62 should be blocked, but p62 did not increase but decreased in cells infected by *T. gondii* at the MOI of 2:1 and 4:1 at 24 h post-infection (Figure 1B). And constant results were observed in BafA1 treated cells infected by *T. gondii* at the MOI of 2:1 for 18 h, 24 h, 36 h, and 48 h (Figure 1C). We speculate that this was because the cells were treated with BafA1 for only 2 h before harvest, and the accumulation of p62 in this short duration was not sufficient to offset the earlier depletion due to the autophagy induced by the parasite infection. Further, this autophagy seems different from the one in IFN-γ-stimulated cells for the recruitment of LC3 to the PVM is less abundant, and a mount of punctate aggregation of LC3 localized near to but not on the parasite PVM (Figure 4; Selleck et al., 2015; Krishnamurthy et al., 2017; Evans et al., 2018; Skariah et al., 2022). These findings lead to an IFN-γ-independent anti-*T. gondii* mechanism through autophagy, which is likely to be different from the IFN-γ-dependent ones for the parasite PV does not appear to be the only target of the autophagy, and other membrane structures near the PVs are also potential ones.

After having found that *T. gondii*-induced autophagy in unstimulated cells may not directly target the parasite PV, we turned our attention to its effect on the nutrient acquisition of the parasite. It has been shown that Rab proteins play important roles in regulating intracellular membrane trafficking (Homma et al., 2021), and for *T. gondii*, hijacking Rab-positive vesicles is also one of the means by which it obtains nutrients from host cells (Coppens et al., 2006; Romano et al., 2013). We examined the expression of several Rabs at the transcriptional level in wild-type and *atg5*^−/−^ HeLa cells since the disruption of *atg5* leads to autophagy deficiency. Compared to wild-type cells, the transcription of *rab11a* decreased in *atg5*^−/−^ cells (Figure 6C). Previous studies implicated the involvement of Rab11A in autophagosomes formation (Longatti et al., 2012), and the down-regulation of the *rab11a* transcription in *atg5*^−/−^ cells could be due to the reduced demand of Rab11A following autophagy deficiency. In wild-type and *atg5*^−/−^ HeLa cells, *rab11a* transcription increased after *T. gondii* infection while the protein abundance did not increase, and even decreased in *atg5*^−/−^ cells, this might be caused by the consumption of Rab11A by the parasite. Compared with wild-type cells, the up-regulation of *rab11a* expression induced by *T. gondii* infection was greater while the abundance of Rab11A was decreased in *atg5*^−/−^ cells (Figures 6B,E). These results indicated that *T. gondii* may capture more Rab11A when host cell autophagy impaired, suggesting that host cell autophagy induced by the parasite infection may be related to the maintenance of host cell cytoplasm Rab11A. The enhancement of autophagic activities induced by *T. gondii* infection was greater in *rab11a*^−/−^ cells than that in wild-type cells, and the intracellular replication of the parasite was slower in this mutant. This not only verified the requirement of *T. gondii* for host cell Rab11A, but also confirmed its link to the parasite-induced autophagy. On the other hand, the absence of Rab11A resulted in more severe autophagy in HeLa cells after *T. gondii* infection, suggesting that there may be other membrane structures besides Rab11a-positive vesicles that are the targets of host cell autophagy induced by *T. gondii* infection. These structures may partially compensate for the Rab11A-positive vesicles required by *T. gondii* and also make it necessary for host cells to prevent the parasite from acquiring them through autophagy.

In addition to host cell proteins, we also investigated the parasite proteins involved in *T. gondii*-induced host cell autophagy. We first addressed secretory and transmembrane proteins. It has been shown that GRA2 involves in the vesicle sequestration of *T. gondii* infected cells (Romano et al., 2017). Here, we showed that knockout of *gra2* in RH∆*ku80* aggravated the autophagy induced by *T. gondii* infection and slowed down the intracellular parasite replication of the parasite (Figure 8), indicating that GRA2 may play a role in combating host cell autophagy. In *T. gondii* infected HeLa cells, the parasite hijacks Rabs-positive vesicles of host cells to obtain nutrients and host cells prevent this process by autophagy. Naturally, *T. gondii* should also have a way to resist this kind of autophagy, and GRA2 is likely to participate in this process. Competing with host cells for Rab11A, or more. In addition to GRA2, we also found other proteins that may be involved in the acquisition of Rab11A by the parasite. ROP18 is an important virulence protein involved in *T. gondii* evading host immunity and is directly related to the virulence of different strains (Etheridge et al., 2014; Shwab et al., 2016; Zhang et al., 2019). GRA16 is one of the earliest *T. gondii* virulence proteins found to be able to regulate host cell processes. The expression of these two proteins was also increased in *rab11a*^−/−^ HeLa cells, further research is needed to find out whether they were also involved and what roles they play in *T. gondii*-induced autophagy. Although the lack of GRA7 did not affect the *T. gondii* obtaining Rab11A-positive vesicles (Romano et al., 2017), the *T. gondii*-induced increase of *gra7* transcripts was significantly greater in *rab11a*^−/−^ HeLa cells than in wild-type cells (Figure 6D), this might be due to the increased compensatory demand for nutrients caused by insufficient Rab11A acquisition as previously suggested (Coppens et al., 2006).

Taken together, we found that without being stimulated by IFN-γ, host cells’ autophagy can be induced by *T. gondii* infection *in vitro*, which can limit intracellular replication of the parasite. Host Rab11A and *T. gondii* GRA2 are likely involved in this process, suggesting a resistance mechanism of host cells to parasite hijacking of membrane structures such as Rab11A-positive vesicles. These findings contribute to a novel understanding of the host immune response to *T. gondii* infection and the associated host–parasite interactions.

## Data availability statement

The raw data supporting the conclusions of this article will be made available by the authors, without undue reservation.

## Author contributions

LP, AD, YY, and YY conceived and supported the study. LP designed and performed the laboratory work. LP, CY, and KS carried out data analysis. XC, GM, and MZ purchased experimental materials and performed a part of the laboratory work. LP wrote the manuscript. CY and AD were in charge of reviewing and editing. All authors contributed to the article and approved the submitted version.

## Funding

This project was funded by the National Natural Science Foundation of China (grant no. 31672543), the Natural Science Foundation of Zhejiang province, China (grant no. LQ21C180002), Zhejiang Province “Sannongliufang” Science and Technology Cooperation Project (grant no. 2020SNLF007).

## Conflict of interest

The authors declare that the research was conducted in the absence of any commercial or financial relationships that could be construed as a potential conflict of interest.

## Publisher’s note

All claims expressed in this article are solely those of the authors and do not necessarily represent those of their affiliated organizations, or those of the publisher, the editors and the reviewers. Any product that may be evaluated in this article, or claim that may be made by its manufacturer, is not guaranteed or endorsed by the publisher.
