# Supplementary material for: Host autophagy limits Toxoplasma gondii proliferation in the absence of IFN-γ by affecting the hijack of Rab11A-positive vesicles
Source: Front Microbiol. 2022 Dec 1;13:1052779. doi: 10.3389/fmicb.2022.1052779 (PMC9751017; doi:10.3389/fmicb.2022.1052779)
Supplement: Supplementary file 1 [file Table_1.docx]

| Name | Sequence (5' to 3') | |
| --- | --- | --- |
| ATG5 lenti-sgF | | CACCGGTTAGCCGGCCTCCCTGCG |
| ATG5 lenti-sgR | | AAACAGTGTGTGCAACTGTCCATC |
| Rab11A lenti-sg1 F | | CACCGAGTGATCTACGTCATCTCA |
| Rab11A lenti-sg1 R | | AAACTGAGATGACGTAGATCACTC |
| GRA2 sgF | | AGTTGGCAGAAAAAGGCTTCACCG |
| GRA2 sgR | | AAACCGGTGAAGCCTTTTTCTGCC |
| GRA2 donorF | | TTTGATTAGATATTGCTTCTTCTCCACATATCGCCTCACATCGAGGTCGACGGTATCGAT |
| GRA2 donorR | | CGGCTTTCCAGCCTGCGCATCACTTTCGTCGTAGTCAACATCCACCGCGGTGGCGGCCGC |
| PCR1 F | | TCGCACGTTGTTTCTCTTCCCA |
| PCR1 R | | GGGTCGATACGTCGCTTCAT |
| PCR2 F | | TCGTGTGACAAAAACGACGC |
| PCR2 R | | TTTCCAGCCTGCGCATCACT |
| PCR3 F | | TCTGACTGTTCATCGCACCC |
| PCR3 R | | TTTCTGCCAGGCCATTAGGG |

**Supplementary Table S1** Primers used in the current study.
