# Supplementary material for: Host autophagy limits Toxoplasma gondii proliferation in the absence of IFN-γ by affecting the hijack of Rab11A-positive vesicles
Source: Front Microbiol. 2022 Dec 1;13:1052779. doi: 10.3389/fmicb.2022.1052779 (PMC9751017; doi:10.3389/fmicb.2022.1052779)
Supplement: Supplementary file 2 [file Data_Sheet_1.docx]

# Supplementary Figure Legends

**FIG S1** *T. gondii* infection increases LC3 II in Vero, HEK293T, and HFF-1 cells. (A) Untreated HEK293T cells and cells challenged with *T. gondii* RH∆*ku80* or ME49 tachyzoites at an MOI of 2:1 were harvested and prepared at 24 h post-infection, and proteins of equal amounts of cell lysates were separated on 15% SDS-PAGE gels. LC3 (16~18 kDa) was detected using rabbit anti-LC3 monoclonal antibody, and Tubulin (~55 kDa) expression was detected using mouse anti-β-tubulin polyclonal antibodies as a loading control. Relative densities of LC3 II were the ratios of the densities of LC3 II to the densities of Tubulin. The relative densities of the negative controls were set as one arbitrary unit whereas those of the treated groups were ratios to the negative controls. Each value is the mean ± SEM of three measurements (*, *P* ≤ 0.05; **, *P* ≤ 0.01; ***, *P* ≤ 0.001; t-tests). (B) HFF-1 cells challenged with *T. gondii* RH∆*ku80* and ME49 strains were collected at 24 h post-infection, and the detection and calculation of relative LC3 II were operated like A. (C) HFF-1 cells challenged with *T. gondii* RH∆*ku80* were harvested at different time points post-infection, and the detection and calculation of relative LC3 II were operated like A. (D) Vero cells challenged with *T. gondii* RH∆*ku80* strain at different MOIs were collected at 24 h post-infection. Relative densities of LC3 II were the ratios of the densities of LC3 II to the densities of LC3 I, each value was the mean ± SEM of three measurements (**, *P* ≤ 0.01; ***, *P* ≤ 0.001; t-tests).

**FIG S2** Autophagosome-like structure increases in *T. gondii* infected Vero and HFF-1 cells. (A) Vero cells were challenged by *T. gondii* RH∆*ku80* tachyzoites at an MOI of 2:1 and fixed by 2.5% glutaraldehyde at 24 h post-infection. (B) HFF-1 cells were challenged by ∆*ku80* tachyzoites at an MOI of 2:1 and fixed by 2.5% glutaraldehyde at 18 h post-infection. (C) Vero cells were challenged by *T. gondii* ME49 tachyzoites at an MOI of 2:1 and fixed by 2.5% glutaraldehyde at 24 h post-infection.

Autophagosome-like membrane structures (As the black arrows show) could be observed in cells infected with *T. gondii*.

**FIG S3** Activated autophagic flux in *T. gondii* infected HFF-1 cells. (A) HFF-1 cells overexpressing mRFP-GFP-LC3 were challenged by *T. gondii* RH∆*ku80* tachyzoites at an MOI of 2:1 and fixed by 4% paraformaldehyde at 24 h post-infection. Nuclei were stained by DAPI. The fluorescence signals of untreated cells (N) and infected cells (∆*ku80*) were collected. Mean density of single fluorescence over the full image range of each merged image was measured after channels splitting, each value is the mean ± SEM of 4 microscope fields (**, *P* ≤ 0.01; t-tests). (B) For Vero cells, no activated autophagic flux in *T. gondii* infected cells (∆*ku80*) and autophagy inducer Torin1 treated cells (Torin1) was observed.

**FIG S4** Transcription of *T. gondii* virulence proteins in *atg5*^−/−^ HeLa cells. (A) Wild-type and *atg5*^−/−^ HeLa cells were harvested at 24 h post-passage, and proteins of equal amounts of cell lysates were separated on 12% SDS-PAGE gels. ATG5 (~32kDa, ~55kDa after combined with ATG12) expression was detected by rabbit anti-ATG5 monoclonal antibody, and Actin (~38 kDa) expression was detected using rabbit anti-Actin monoclonal antibody as a loading control. (B) HeLa cells were challenged by *T. gondii* RH∆*ku80* tachyzoites at an MOI of 2:1, and total RNA and protein were extracted using TRIzol at 24 h post-infection. The relative mRNA abundance of *T. gondii gras and rops* was quantified by qPCR, and *Tgtubulin* was used as a standard to normalize all data by 2^−ΔΔCT^ methods. Each value is the mean ± SEM of three experiments.

**FIG S5** Identification of *rab11a*^−/−^ HeLa cells and *T. gondii* RH∆*ku80*∆*gra2*. (A) Wild-type and *rab11a*^−/−^ HeLa cells were harvested at 24 h post-passage, and proteins of equal amounts of cell lysates were separated on 12% SDS-PAGE gels. Rab11A (~24kDa) expression was detected using rabbit anti-Rab11A monoclonal antibody, and Tubulin (~55 kDa) expression was detected using mouse anti-β-tubulin polyclonal antibodies as a loading control. (B) Construction and identification of *T. gondii* RH∆*ku80*∆*gra2*.
