# Supplementary figures and images for: Host autophagy limits Toxoplasma gondii proliferation in the absence of IFN-γ by affecting the hijack of Rab11A-positive vesicles
Source: Front Microbiol. 2022 Dec 1;13:1052779. doi: 10.3389/fmicb.2022.1052779 (PMC9751017; doi:10.3389/fmicb.2022.1052779)

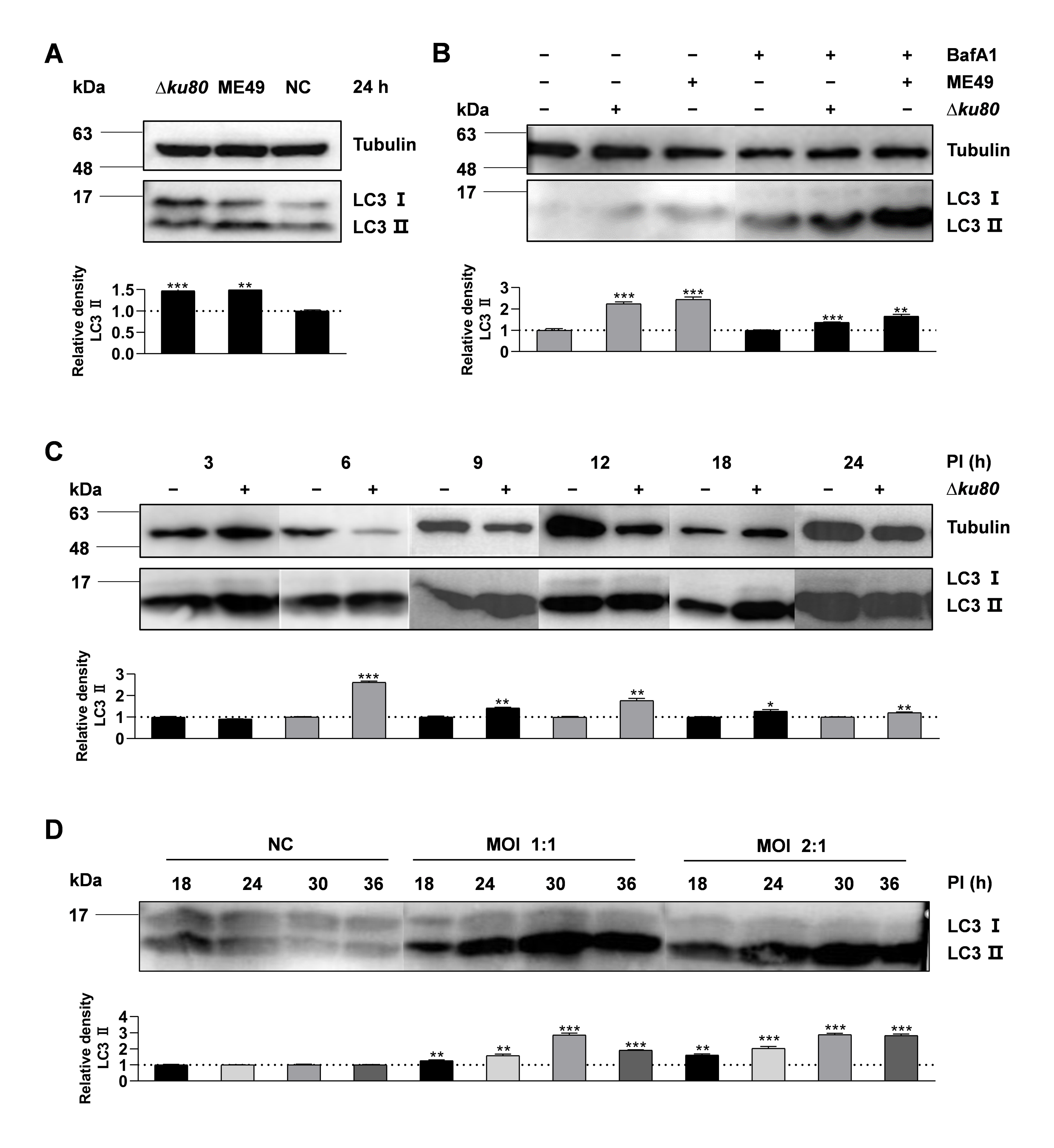

Supplement: Supplementary file 3 [file Image_1.TIF]

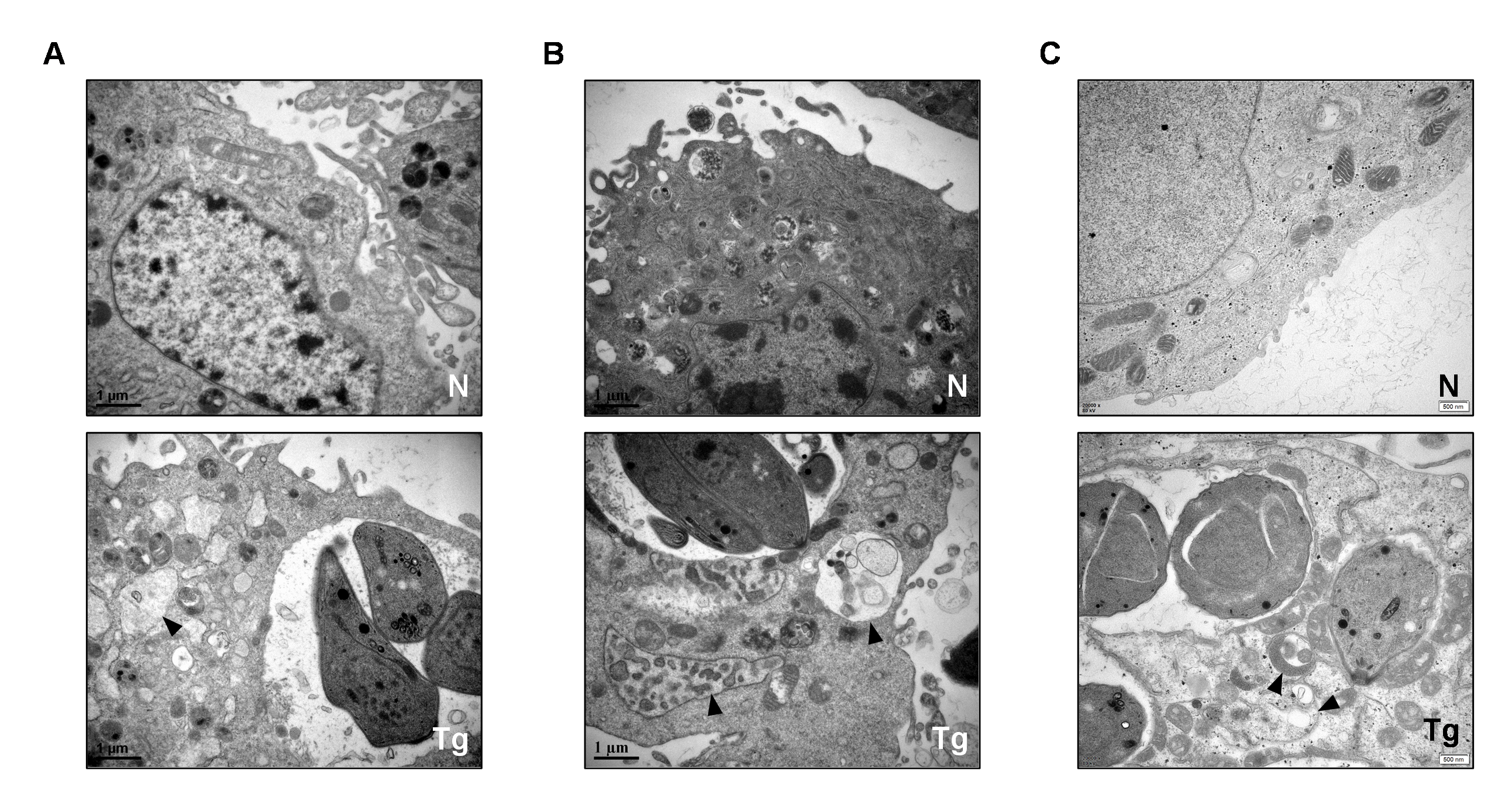

Supplement: Supplementary file 4 [file Image_2.TIF]

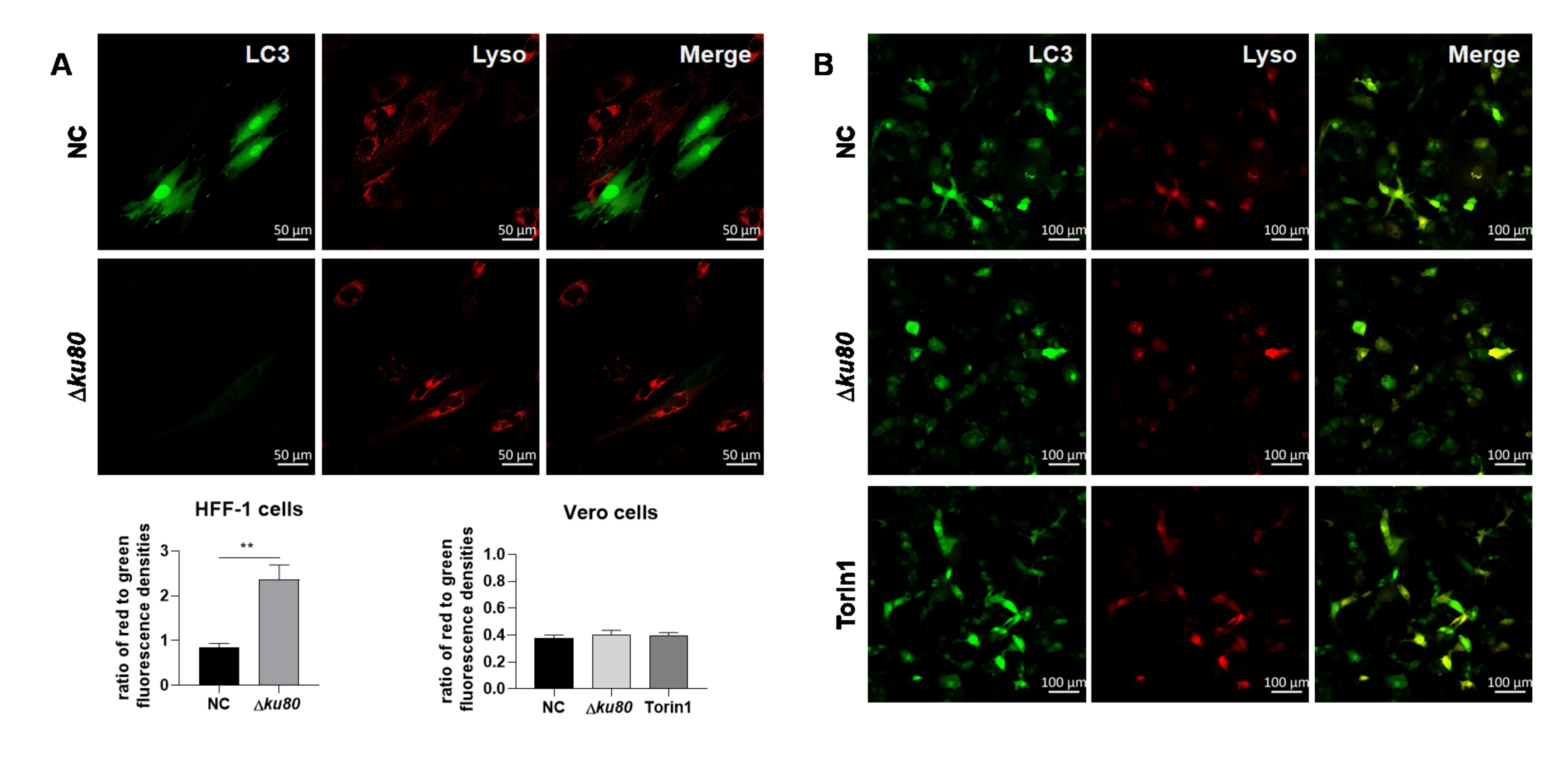

Supplement: Supplementary file 5 [file Image_3.TIF]

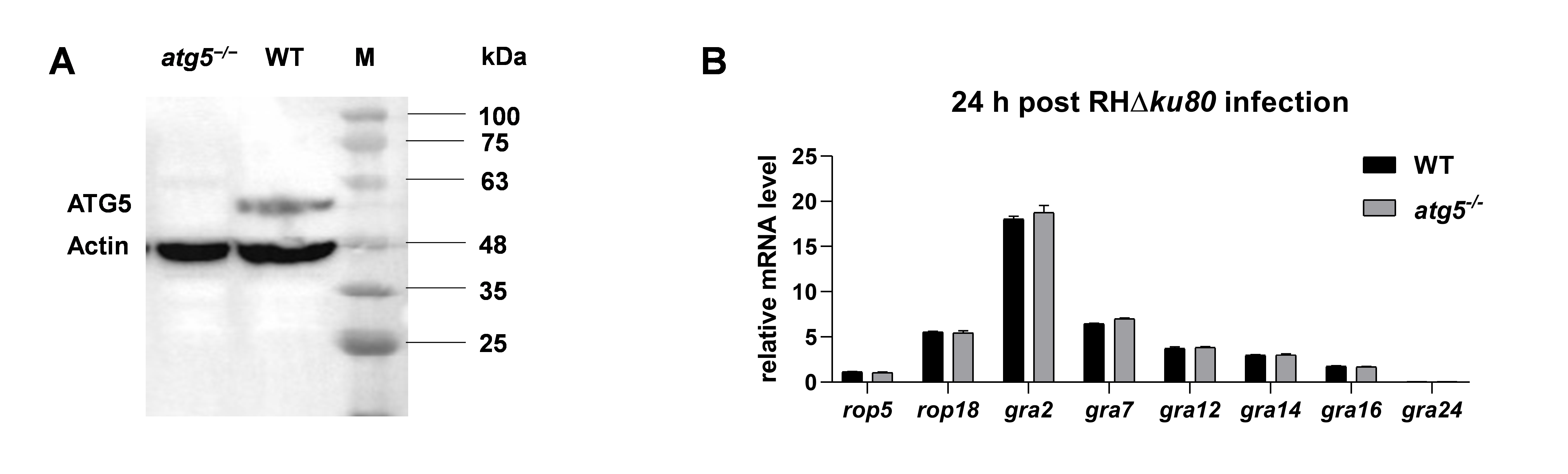

Supplement: Supplementary file 6 [file Image_4.TIF]

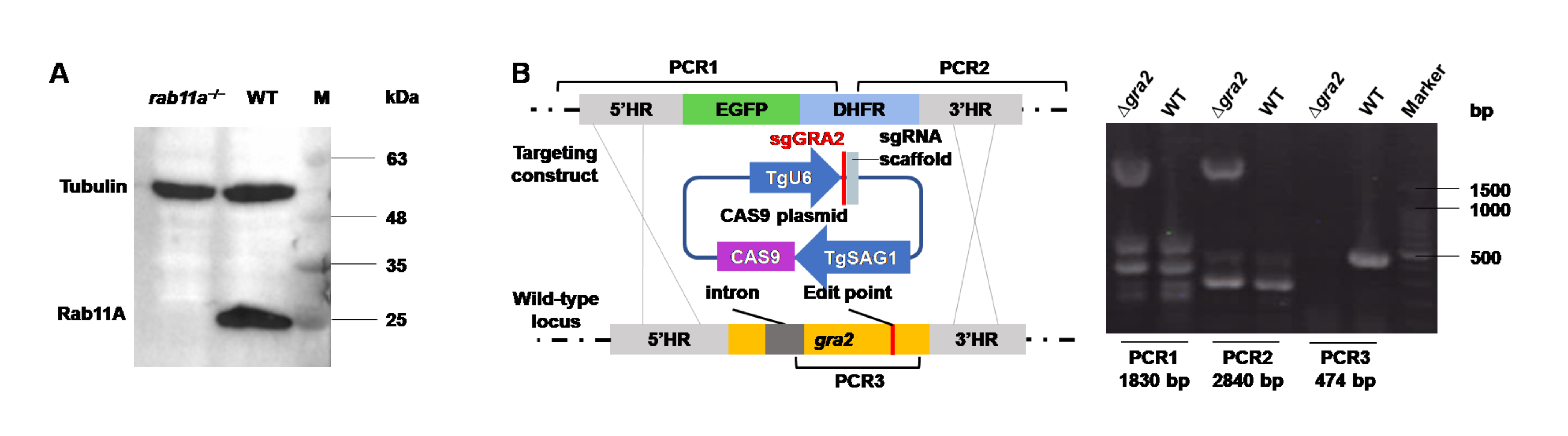

Supplement: Supplementary file 7 [file Image_5.TIF]
